# Multifunctional Guest-Hosting Triple-Stranded Helicates: From Anion Recognition to Quantum Information Applications

**DOI:** 10.1021/acs.accounts.6c00020

**Published:** 2026-02-26

**Authors:** Abinash Swain, Valentin Novikov, Olivier Roubeau, Leoní A. Barrios, Guillem Aromí

**Affiliations:** # Departament de Química Inorgànica i Orgànica, 16724 Universitat de Barcelona, Diagonal 645, 08028 Barcelona, Spain; $ Institute of Nanoscience and Nanotechnology of the University of Barcelona (IN2UB), 08028 Barcelona, Spain; † Instituto de Nanociencia y Materiales de Aragón, CSIC and Universidad de Zaragoza, 50009 Zaragoza, Spain

## Abstract

The growing field of coordination supramolecular chemistry constitutes a fruitful avenue for accessing a variety of multifunctional materials with a range of applications. Their versatility is enhanced if they have the ability to encapsulate guest molecules, opening opportunities for host/guest synergies. One of the most paradigmatic categories of such assemblies is coordination supramolecular helicates, which exhibit a central cavity for the potential allocation of small species, provided that their symmetry and volumes are compatible. The presence of noncovalent interactions (NCIs) between host and guest strongly contributes to the thermodynamic stability of these edifices, sometimes giving rise to a template effect. All those features are exploited for the case of triple-stranded helicates, which are predictably obtained from reactions of metal ions that adopt an octahedral coordination geometry with ligands made of two chelating moieties sufficiently separated by a spacer. The properties of the cavity of the helicate can be tuned by adjusting the central spacer of the ligand, which in turn, may incorporate functionalities facilitating NCIs with potential guests, such as hydrogen bonds. In this manner, a collection of pyrazolylpyridine (or -quinoline) ligands (L) has given rise to a large family of (G@[M_2_L_3_])^*n*+^ species (where G represents various guests), in which the encapsulated entities are firmly held in place by [N–H···G] hydrogen bonds. These assemblies can thus be employed for the selective recognition of anions or small coordination complexes, capitalizing on the specific architecture of the ligand strands. Furthermore, they have opened a plethora of possibilities for the investigation of synergic multifunctionality. The host can be made to exhibit molecular switching behavior (for example, spin-crossover, SCO, if M = Fe^II^) or single-ion magnet (SIM) behavior (if M = Co^II^) while the guest has been exploited to tune these properties or to incorporate new ones. More recently, anionic coordination complexes such as these from the series [M­(ox)_3_]^3–^ (“ox” being the oxalate anion and M = Fe, Cr, Al, Ru) have been efficiently trapped inside the metallo-helices. This has unveiled unprecedented phenomena resulting from encapsulation, such as the first manifestation of SIM behavior for Cr^III^ or the enhancement of the quantum coherence of a molecular qubit when acting as the guest. This family has been expanded with the inclusion of the anilate analogues of oxalates, opening unlimited options for multiproperty explorations (such as photophysical, redox chemistry, radical generation, etc.). More recently, within this group of systems, the guest has been employed as a template to selectively assemble specific combinations of two different ligands in the form of G@[M_2_L_*x*_L′_(3–*x*)_]^*m*+^ heteroleptic helicates, thus leading to a further opportunity of function tunability and enhancement. In this Account, we survey this and other related types of host/guest assemblies and place them in the general context of triple-stranded supramolecular helicates while assessing their impact in fields like molecular magnetism, quantum technologies, or anion recognition.

## Graphical abstract

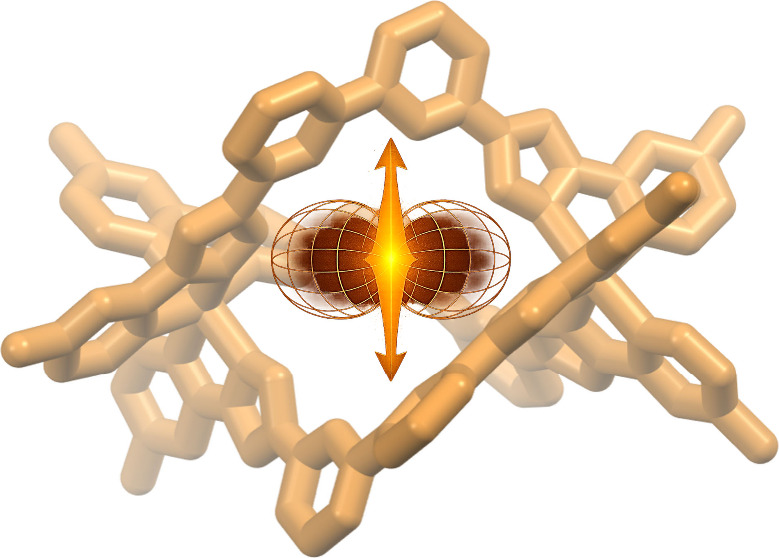

## Key References

- Darawsheh, M.; Barrios, L. A.; Roubeau, O.; Teat, S. J.; Aromí, G. Encapsulation of a Cr^III^ Single-Ion Magnet within an Fe^II^ Spin-Crossover Supramolecular Host. Angew. Chem., Int. Ed. 2018, 57, 13509–13513.10.1002/anie.20180725630161280 ^1^ First encapsulation of an [M­(ox)_3_]^3–^ complex and unprecedented discovery of single-ion magnet behavior for Cr­(III).
- Capó, N.; Barrios, L. A.; Cardona, J.; Ribas-Ariño, J.; Teat, S. J.; Roubeau, O.; Aromí, G. The template effect of a SiF_6_^2–^ guest drives the formation of a heteroleptic Fe­(II) coordination helicate. Chem. Comm. 2022, 58, 10969–10972.36089837 10.1039/d2cc04559a^2^ Discovery of the template effect by a guest for the realization of heteroleptic triple-stranded metallo-supramolecular helicates.
- Aleshin, D. Y.; Diego, R.; Barrios, L. A.; Nelyubina, Y. V.; Aromí, G.; Novikov, V. V. Unravelling of a [High-spin—Low-spin]↔[Low-spin—High-spin] Equilibrium in Spin-Crossover Iron­(II) Dinuclear Helicates Using Paramagnetic NMR Spectroscopy. Angew. Chem., Int. Ed. 2022, 61, e202110310.10.1002/anie.20211031034757659 ^3^ First evidence of Fe­(II) spin state permutation within a dinuclear molecule, obtained from NMR studies on a helicate.
- Swain, A.; Barrios, L. A.; Nelyubina, Y. V.; Teat, S. J.; Roubeau, O.; Novikov, V. V.; Aromí, G. Encapsulation Enhances the Quantum Coherence of a Solid-State Molecular Spin Qubit. Angew. Chem., Int. Ed. 2025, e202510603.10.1002/anie.202510603PMC1251869640888488 ^4^ Demonstration that supramolecular encapsulation allows the increase of the coherence of a spin molecular qubit in a solid lattice.

## 1 Introduction

A commonly cited definition of supramolecular chemistry describes it as *“the chemistry beyond the molecule”*.^5,6^ It encompasses the structures and phenomena resulting from the association of molecular species through intermolecular interactions, most typically but not exclusively,^7^ hydrogen bonds,^8–13^ π···π ^14–20^ or anion···π interactions.^21–25^ The concepts of this discipline are crucial for most processes and organisms of the living world,^26^ but are also important in other fields^27^ such as materials sciences,^28,29^ nanotechnology,^14,30–33^ environmental chemistry,^29^ catalysis,^34–37^ or analytical chemistry.^38^ In this context, *coordination supramolecular chemistry* can be considered a part of this discipline, but with peculiarities. On the one hand, the categorization as supramolecular of the complexes arising from this field is sustained by the fact that the structure of the ligands and the information contained in metals’ geometric preferences can be engineered for the programmed construction of complex so-called metallosupramolecular architectures^39^ in the form of metallacycles,^24,36,40–46^ grids,^47–49^ helicates^50,51^ or metallacages,^36,42,44,45,52^ among other arrangements. On the other hand, the energy of the coordination bond approximately spans the gap between that of covalent bonds and that of intermolecular forces. Therefore, for the most labile coordination bonds, these complexes are the result of constitutional dynamic chemistry (CDC)^53–57^ process among their components, as is inherent to genuine supramolecular chemistry. Interestingly, we often find well-defined, stable metallosupramolecular species engaging in generic processes *“beyond the molecule”*, by undergoing weak interactions with other molecules.^24,43,58^ These phenomena are called *“Hierarchical self-assembly (HSA)”* processes,^58^ a frequent example being host–guest systems involving coordination receptors.^37,45,59–62^ They constitute an opportunity to exploit multifunctionality at the molecular scale, for example, to realize catalysis with a confined substrate.^63–65^ The encapsulation is most commonly found with metallacages,^37,61,64,65^ but also with metallacycles^61,63^ and helicates.^62,66–69^

Metallosupramolecular helicates are coordination complexes formed by self-assembly of metal ions and oligodentate ligands.^50,51^ The geometry preferences of both components conform an “algorithm” that is expressed through a chemical reaction.^54,62^ The stereochemistry adopted by the metal center upon chelation defines the handedness of the helicate. Most metallohelicates are dinuclear, but can also contain more than two metal nodes.^70–74^ The majority of them are triple-stranded,^50,51,75–77^ reflecting the fact that di- or trivalent metals with octahedral coordination geometry are bound by three bidentate chelating moieties, forming a local helical motif (Figure 1). The chelating groups of the ligands must be separated by a spacer enabling the formation of the supramolecular helix, also determining the size of the cavity at the center of the helicate for the allocation of potential guests.

**Figure 1:**
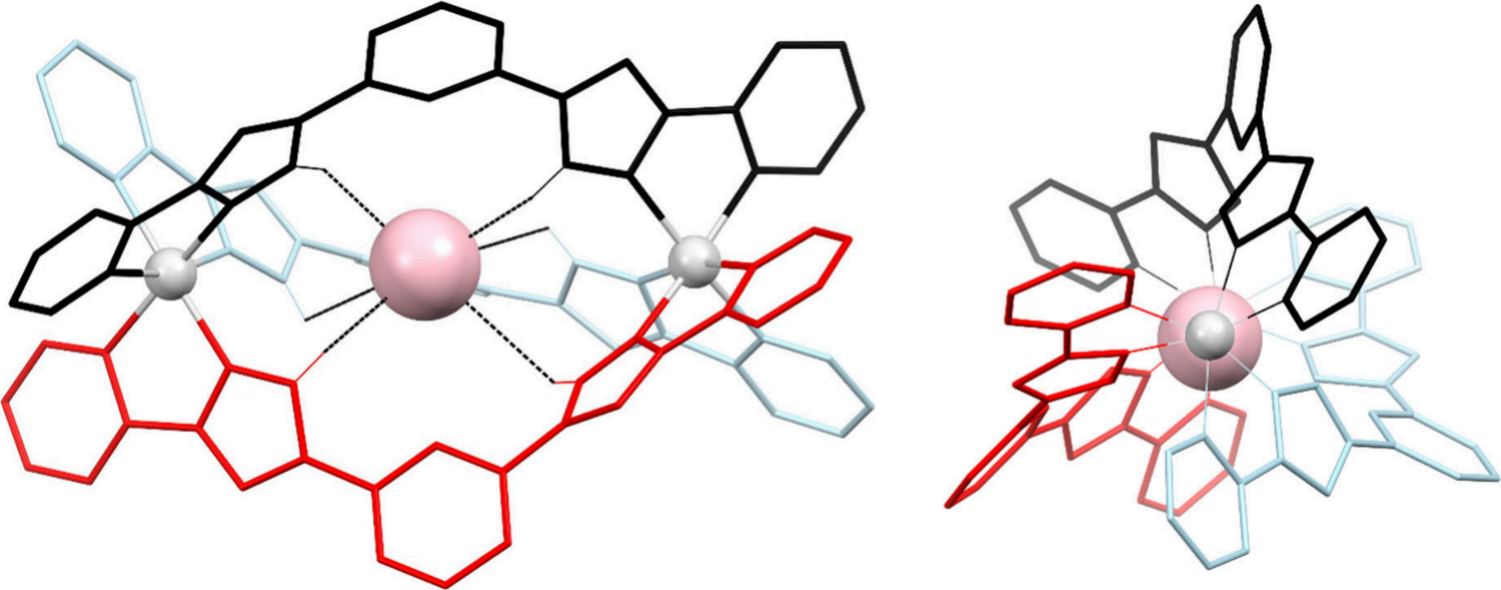
Two perpendicular views of a triple-stranded dinuclear helicate [M_2_L_3_]^4+^ (M = Ni^2+^, Co^2+^, Fe^2+^, or Zn^2+^), where L features two pyrazolylpyridine chelating groups. Each ligand is shown in a different color. The central cavity is occupied by a halide ion (Cl^–^ or Br^–^). Only N–H hydrogen atoms are displayed in the left figure, participating hydrogen bonds shown as dashed lines.

Some chelates used to generate coordination helicates are hydroxamic acid moieties,^78,79^ oxamates,^73,74^ hydroxyquinolines,^69^ catechols,^67,68,80^ β-diketones,^77,81–84^ pyridylmethaimines,^85^ bipyridine,^66^ or dithiolates.^86^ Depending on the charge of the components, the helicates may be cationic, neutral or anionic. Most reported coordination helicates do not exhibit any guests. For one to be present, the volume and symmetry of the cavity must fulfill the geometric requirements of the hosted species while both parties must present the ability to mutually establish intermolecular interactions. Cationic guests are usually fixed by cavity atoms acting as Lewis bases.^51,62,69,87,88^ Encapsulated neutral organic molecules may benefit from π···π or C–H··· π interactions, or hydrogen bonds.^67^ Anionic guests can act as acceptors in hydrogen bridges with the helicate’s ligands.

In this account, we describe the multifunctional properties of a family of host–guest dinuclear triple-stranded helicates formed with *bis*-pyrazolylpyridine (or -quinoline) ligands that incorporate central spacers of variable length (Figure 2). Upon reaction with divalent metal ions (Fe^2+^, Co^2+^, Ni^2+^, Zn^2+^, Cu^2+^), most of these ligands undergo a self-assembly process producing thermodynamic helical aggregates with formula [M_2_L_3_]^4+^, with notable exceptions discussed below. The supramolecular structure is always obtained with an encapsulated guest as a (G@[M_2_(L)_3_])^*n*+^ cation (G = guest), the empty capsules remaining highly elusive. All guests identified are anionic Lewis bases that serve as acceptors for six hydrogen bonds with the N–H groups of the ligand strands, which point toward the interior of the cavity.

**Figure 2:**
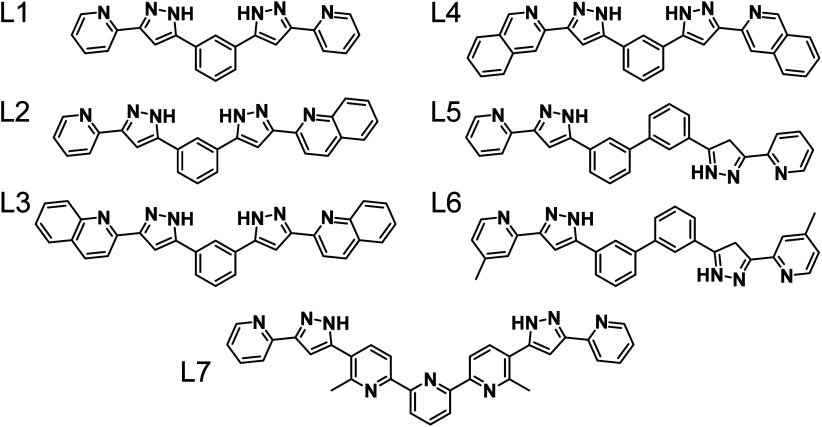
*bis*-Pyrazolylpyridine (or -quinoline) ligands discussed in this Account.

## 2 Halide Encapsulation

The ability of *bis*-pyrazolylpyridine ligands to form [M_2_L_3_]^4+^ helicates was first demonstrated for L1 ^89^ (Figure 2), which upon reaction with MX_2_ (M = divalent metal ion; X = Cl^–^, Br^–^) systematically forms crystals with (X@[M_2_(L1)_3_])^3+^ as cation (Figure 1). The single crystal X-ray diffraction (SCXRD) was obtained for M = Fe^2+^,^89^ Co^2+^,^90^ Ni^2+^,^91^ and Zn^2+^,^90^ in most cases for both guests, Cl^–^ and Br^–^. The use of a second anion (BF_4_^–^ or PF_6_^–^), different from the guest, facilitates the crystallization process and causes its presence in the lattice. While encapsulation of X^–^ seems crucial to isolate the helicate, the empty host is almost always detected by mass spectrometry (MS), since this technique often unveils nonthermodynamically favored species.^89–91^ The halide is fixed by six [X···H–N] hydrogen bonds with the conveniently oriented N–H groups of the ligands (Figure 1). Despite many attempts,^89^ it has not been possible to encapsulate F^–^ or I^–^ anions, which suggests that the [M_2_(L1)_3_]^4+^ host can selectively scavenge Cl^–^ and Br^–^ anions (see Table 1 for the empty volume inside all helicate types discussed in this review, calculated with the program MoloVol^92^). A synthetic strategy to encapsulate larger guests is to increase the length of the central spacer of the ligand strands (see below, Table 1). In all cases, the stability and idealized *D*_*3*_ symmetry of the helical assembly was established by ^1^H NMR (Figure 3),^89–91^ which also provides evidence that the guest remains encapsulated in solution.^3,91^ This technique, together with MS, was used to establish for the case of (X@[Ni_2_(L1)_3_])^3+^, higher affinity for Cl^–^ than for Br^–^.^91^ The energy required to replace one guest by the other was estimated to be 19.8 kcal·mol^–1^ by DFT calculations on the geometry-optimized species.^91^

**Figure 3:**
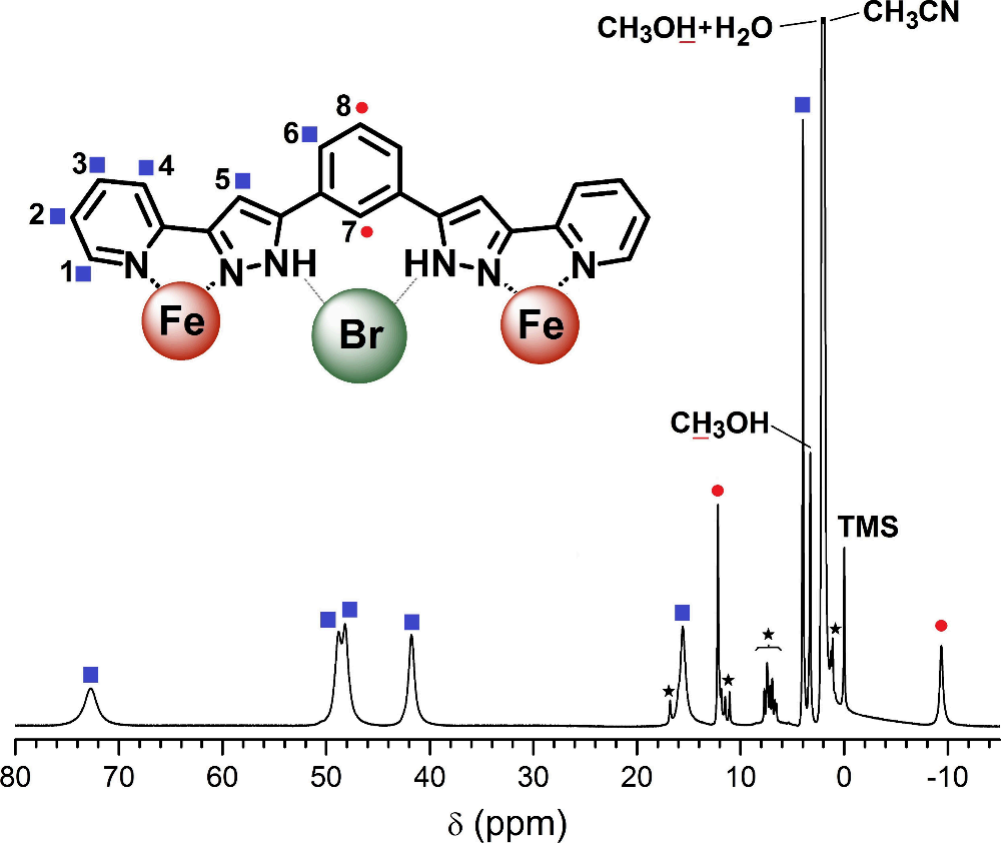
Room temperature aramagnetic 300 MHz ^1^H NMR spectrum of the cation (Br@[Fe_2_(L1)_3_])^3+^ in *d*-MeCN, evidencing its stability and *D*_*3*_ symmetry in solution, the latter resulting from a [HS-LS] ↔ [LS-HS] fast exchange process. The NH proton is probably not observed due to H–D exchange. The asterisks are a minor species with lower symmetry (see text). The inset shows the ligand coordination mode and symmetry in solution.

**Table 1:** Calculated Empty Cavity Volumes for the Various Helicate Types in This Paper

Compound	Cavity Volume (Å^3^)^a^/Type
Cl@[Ni_2_(L1)_3_]Cl(PF_6_)_2_	18/isolated
Br@[Ni_2_(L1)_3_]Br(PF_6_)_2_	21/isolated
Cl@[Fe_2_(L1)_3_]Cl(PF_6_)_2_	17/isolated
Br@[Fe_2_(L1)_3_]Br(PF_6_)_2_	20/isolated
SO_4_@[Ni_2_(L1)_2_(L6)](BF_4_)_2_	52/tunnel
SiF_6_@[Fe_2_(L1)(L5)_2_](PF_6_)_2_	115/tunnel
(ClO_4_)@[Fe_2_(L5)_3_](ClO_4_)_3_	148/tunnel
[Fe(ox)_3_]@[Fe_2_(L5)_3_]BF_4_	130/tunnel
[Fe(ani)_3_]@[Fe_2_(L5)_3_]BF_4_	162/tunnel

a Calculated with the program MoloVol.^92^

The family of compounds containing the cations (Cl@[Co_2_(L1)_3_])^3+^, (Cl@[Zn_2_(L1)_3_])^3+^ and (Cl@[CoZn­(L1)_3_])^3+^, was used to study the SIM behavior of Co^2+^ in a rare coordination geometry, in between trigonal prismatic and octahedral.^90^ This was carried out through solid-state static and dynamic magnetic susceptibility measurements and EPR spectroscopy, revealing *S* = 3/2 Co^2+^ centers with Ising-type anisotropy and slow relaxation of the magnetization under applied constant magnetic fields. The absence of any bias on the individual behavior of each Co^2+^ center caused by the presence of two such ions in the molecule was confirmed from studying the heterometallic analogue (Cl@[CoZn­(L1)_3_])^3+^. The latter was obtained as the quasi-exclusive dopant within the crystalline molecular alloy (Cl@[CoZn­(L1)_3_])_0.19_(Cl@[Zn_2_(L1)_3_])_0.81_Cl­(PF_6_)_2_.^90^ The analogous behavior in solution was confirmed through variable temperature paramagnetic ^1^H NMR.

## 3 Spin-Crossover of (G@[Fe_2_L_3_])^3+^ Helicates: First Evidence of [LS-HS] ↔ [HS-LS] Equilibrium in Molecules

It is established that pyrazolyl-pyridine chelating ligands are prone to engender spin-crossover (SCO) properties^93^ when coordinating to Fe^2+^ ions,^94^ therefore, most (G@[Fe_2_L_3_])^*n*+^ complexes are expected to exhibit this behavior (see Table 2 for a summary of the main SCO parameters of all the reviewed compounds). This suggests possible synergies between SCO-active hosts and functional guests. Even the simple halide guests in the assemblies (X@[Fe_2_(L1)_3_])^3+^ (X^–^ = Cl^–^, Br^–^) show unprecedented phenomena. As solids, these complexes exhibit a mixed-spin state over a wide temperature range (2 K to above 200 K).^89^ This is indicated by temperature-dependent magnetic susceptibility (χ) data (Figure 4), while variable temperature SCXRD confirms that at these temperatures, one Fe^2+^ center of the helicate is in the high-spin state (HS, *S* = 2) while the other one is in the low-spin state (LS, *S* = 0), i.e. being a rare example of an ordered molecular mixed-spin state, with each molecule of the lattice being [LS–HS].^95^ Crystallography is informative because the average Fe–N distance of Fe^2+^ ions coordinated by six N atoms increases by ≈12% upon LS to HS state transition (Figure 4).^96,97^ The LS Fe^2+^ center undergoes a SCO if the temperature is increased further, leading to the [HS–HS] system. This transition occurs ∼40 K lower for the Br^–^ guest than for Cl^–^, as detected both magnetically and structurally (Figure 4) and confirmed by calorimetric measurements.^89^ Considering that the pyrazolylpyridine rings can be functionalized, there are ample opportunities to fine-tune the SCO properties of these systems (see below).

**Figure 4:**
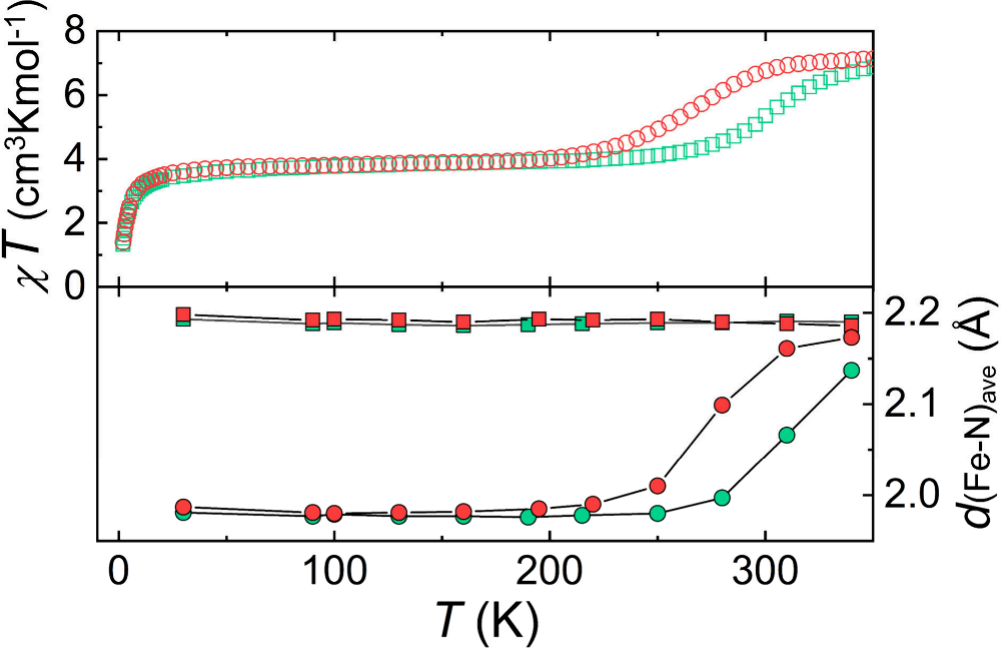
Temperature dependence (top) of *χT* under an applied magnetic field for compounds (X@[Fe_2_(L1)_3_])­X­(PF_6_)_2_ (X^–^ = Cl^–^, green; Br^–^, red) and (bottom) of the average Fe–N distances of their Fe^2+^ centers, as determined through SCXRD (same colors).

**Table 2:** SCO Parameters of All the Fe­(II) Helicates Reviewed Here

Compound	*T*_1/2_ (K)	Σ^a^	Θ^b^	Guest
Cl@[Fe_2_(L1)_3_]Cl(PF_6_)_2_	305^c^	58.80/115.77^d^	189.71/388.66^d^	Cl^–^
		90.74/109.06^e^	281.28/359.31^e^	
Br@[Fe_2_(L1)_3_]Br(PF_6_)_2_	265^c^	61.23/113.62^d^	197.76/363.65^d^	Br^–^
		95.52/106.94^e^	295.26/337.78^e^	
(Cl@[Fe_2_(L1)_3_])^3+^	302,^c^ ^,^ ^f^ 278^c^ ^,^ ^g^	n/a	n/a	Cl^–^
(Br@[Fe_2_(L1)_3_])^3+^	264,^c^ ^,^ ^f^ 228^c^ ^,^ ^g^	n/a	n/a	Br^–^
ClO_4_@[Fe_2_(L5)_3_](ClO_4_)_3_	298^h^	61.93/68.74^i^	197.28/208.21^i^	ClO_4_^–^
ClO_4_@[Fe_2_(L6)_3_](ClO_4_)_3_	321^h^	59.26/66.33^i^	192.44/195.28^i^	ClO_4_^–^
SiF_6_@[Fe_2_(L1)(L5)_2_](PF_6_)_2_	175^c^	93.19/98.25^d^	290.68/306.51^d^	SiF_6_^–^
SiF_6_@[Fe_2_(L1)(L5)_2_](BF_4_)_2_	143^c^	76.31/99.25^d^	239.29/312.34^d^	SiF_6_^–^
SiF_6_@[Fe_2_(L1)(L6)_2_](PF_6_)_2_	169^c^	70.38/97.24^d^	226.53/295.38^d^	SiF_6_^–^
[Cr(ox)_3_]@[Fe_2_(L5)_3_]BF_4_	200^j^	59.90/61.71^i^	182.99/201.39^i^	[Cr(ox)_3_]^3–^
		58.18/88.26^d^	182.99/278.40^d^	
[Fe(ox)_3_]@[Fe_2_(L5)_3_]BF_4_	310^j^	64.69/69.77^i^	198.73/206.91^i^	[Fe(ox)_3_]^3–^
		62.13/83.19^d^	185.38/263.26^d^	
[Fe(ani)_3_]@[Fe_2_(L5)_3_]BF_4_	335/400^j^	43.13/60.71^i^	150.34/200.66^i^	[Fe(ani)_3_]^3–^
		80.69/66.48^e^ ^,^ ^k^	254.03/215.58^e^ ^,^ ^k^	

a Σ is the sum of the deviation away from 90° of the 12 possible cis-NFeN bite angles (Σ = Σ_*i* = 1_^12^|90 – α_*i*_|).

b Θ gauges the twist away from perfect octahedral symmetry toward a trigonal prismatic symmetry (Θ = Σ_*i* = 1_^24^|60 – β_*i*_|).

c [LS–HS] to [HS–HS].

d LS/HS format.

e HS/HS format.

f In methanol solution.

g In acetonitrile solution.

h [LS–LS] to [HS–HS].

i LS/LS format.

j [LS–LS] to [LS–HS].

k For the less distorted metal, the conversion is not complete.

Variable temperature paramagnetic ^1^H NMR unveiled unique SCO properties of the (X@[Fe_2_(L1)_3_])^3+^ assemblies.^3^ At high temperatures (260–320 K), the spectra are consistent with molecular *D*_3_ symmetry (nine signals), arising from two fast dynamic processes, the spin-switching processes of the type [LS–HS] ↔ [HS–HS] together with a [LS–HS] ↔ [HS–LS] exchange of the molecules lying in the mixed-spin state. Below 220 K, the number of signals is larger, consistent with lower symmetry (*C*_3_) following the retardation of the [LS–HS] ↔ [HS–LS] process below the NMR time-scale. In this regime, the system follows the Curie law, confirming that all the molecules remain in the mixed spin-state. The analysis of the data provided for the first time experimental evidence of a [HS–LS] ↔ [LS–HS] equilibrium, here characterized near 220 K with a rate in the order of milliseconds.^3^ Since two protons of the phenylene spacer (7 and 8 in Figure 3) are magnetically not affected by the exchange at all temperatures, their thermal chemical shift dependence was analyzed to establish the SCO curves for both (X@[Fe_2_(L1)_3_])^3+^ species. In both, methanol and acetonitrile, the SCO of the Br^–^ derivative is shifted by 40 K or more to colder temperatures compared to the Cl^–^ one, as observed in the solid state and strongly indicating that the guest encapsulation and its effect on the SCO persist in solution (Table 2).^3^ This unprecedented description of the dynamics of the [LS–HS] ↔ [HS–LS] process in a molecule opens the door to study a molecular “half-cell” double-dot quantum cellular automata (QCA), based on magnetic interactions, rather than Coulombic ones.^98^ The above discoveries confirm the (G@[Fe_2_L_3_])^3+^ or related assemblies as versatile objects to realize synergies between SCO and other functional properties borne by the ligand strands^99^ or by the encapsulated guest.

## 4 Guest-Directed Strand Composition

Because of the selectivity of the cavity, the (X@[Fe_2_(L1)_3_])^3+^ assemblies are accessible for X^–^ = Cl^–^ and Br^–^ but not for F^–^ or I^–^ (presumably too small and too large, respectively; Table 1). When the size of the central spacer in L is increased (e.g., moving from L1 to L5), a larger polyatomic guest may be encapsulated, such as ClO_4_^–^ (Figure 5), while maintaining the ability to form [N–H···O–Cl] hydrogen bonds. Thus, SCXRD structures were obtained for compounds ClO_4_@[Fe_2_(L′)_3_]­(ClO_4_)_3_ (L′ = L5 or L6),^2^ ClO_4_@[Co_2_(L6)_3_]­(ClO_4_)_3_,^100^ and ClO_4_@[Ni_2_(L6)_3_]­(ClO_4_)_3_.^101^ The ferrous compounds exhibit distinct SCO properties (Table 2), whereas the Co one features SIM behavior under an external magnetic field. The magnetic relaxation of the [(Co^2+^)_2_] system was investigated via dynamic magnetization measurements and specific heat to discover a phonon bottleneck phenomenon with two different rates, presumably associated with two different vibronic energy regimes, one for the host and one for the guest.^100^

**Figure 5:**
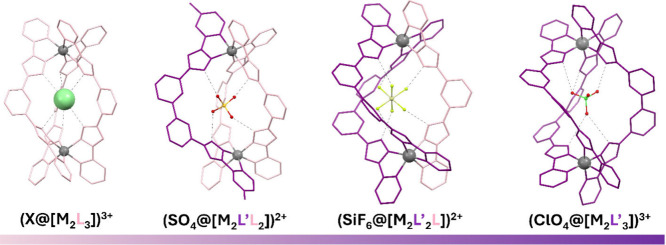
Structures of the supramolecular cations (X@[M_2_(L)_3_])^3+^ (X^–^ = Cl^–^, Br^–^), (SO_4_@[M_2_L′L_2_])^2+^, (SiF_6_@[M_2_L′_2_L])^2+^, and (ClO_4_@[M_2_L′_3_])^3+^ (L = L1; L′ = L5, L6; M = Fe^2+^, Co^2+^, Ni^2+^, Zn^2+^).

An unprecedented characteristic of this family of compounds is that certain guests can template the assembly of heteroleptic triple-stranded helicates with specific ligand combinations. Thus, the mixing of L1 and L5 with Fe­(BF_4_)_2_ in MeOH with an excess of Bu_4_NPF_6_ led unexpectedly to crystals of SiF_6_@[Fe­(L5)_2_(L1)]­(PF_6_)_2_ (Figure 5). Silicon not being among the reagents, the fluorosilicate guest was deduced to form *in situ* from the transfer of BF_4_^–^ fluoride to the silica (SiO_2_) glass of the reaction tubes.^2^ Presumably, the SiF_6_^2–^ anion fits perfectly within the cavity generated by this combination of ligands stabilized by six [N–H···F–Si] hydrogen bonds. Therefore, a strong host–guest affinity possibly drives the extraction of Si^4+^ from the glass. The compound is obtained in higher yield introducing stoichiometrically (Bu_4_N)_2_SiF_6_, also using plastic containers as reaction vessel. NMR spectroscopy allows one to establish the persistence of the supramolecular ensemble in solution. The ^1^H NMR spectrum confirms the *C*_2_ symmetry of the helicate, whereas the stability of the assembly and the exclusive selectivity of SiF_6_^2–^ in front of PF_6_^–^ or BF_4_^–^ is corroborated with ^19^F NMR.^2^ Despite persistent attempts, the analogous compound encapsulating PF_6_^–^ could never be obtained. The above procedure can be replicated to prepare the counterparts SiF_6_@[Fe­(L6)_2_(L1)]­(PF_6_)_2_,^2^ SiF_6_@[Co­(L6)_2_(L1)]­(PF_6_)_2_,^100^ SiF_6_@[Ni­(L6)_2_(L1)]­(PF_6_)_2_ and SiF_6_@[Zn­(L6)_2_(L1)]­(PF_6_)_2_.^101^ The combination of two “short” (L1) and one “long” (L5 or L6) ligand can also be accessed if SO_4_^2–^ is used as guest, which selectively promotes the exclusive assembly SO_4_@[Ni­(L6)­(L1)_2_]­(BF_4_)_2_ (Figure 5).^101^ The selectivity of different guests for the [Ni­(L6)_2_(L1)]^4+^ host was investigated by means of DFT calculations. The energy of the helicate self-assembly and encapsulation of halides, NO_3_^–^, ClO_4_^–^, BF_4_^–^, PF_6_^–^, SO_4_^2–^, ZrF_6_^2–^, SiF_6_^2–^, and AlF_6_^3–^ was computed and compared with the formation of empty helicate. It was found that the encapsulation of AlF_6_^3–^ is the most favored, followed by SO_4_^2–^, SiF_6_^2–^ and ZrF_6_^2–^·^101^ The ability to combine two different ligands within a triple-stranded helicate expands the options for incorporating various functionalities into the supramolecular assembly. For example, the combination of the asymmetric ligand L2 with L6 in a reaction with Ni­(PF_6_)_2_ and (Bu_4_N)_2_SiF_6_ conduces, predictably, to the compound SiF_6_@[Ni­(L6)_2_L2]­(PF_6_)_2_ (Figure 6), where each Ni^2+^ exhibits a different coordination environment. Considering that Ni^2+^ complexes are possible realizations of spin-based qubits for quantum technologies, access to complexes containing two different and weakly coupled Ni^2+^ *S* = 1 centers is of interest for the search of two-qubit conditional quantum gates.^102,103^

**Figure 6:**
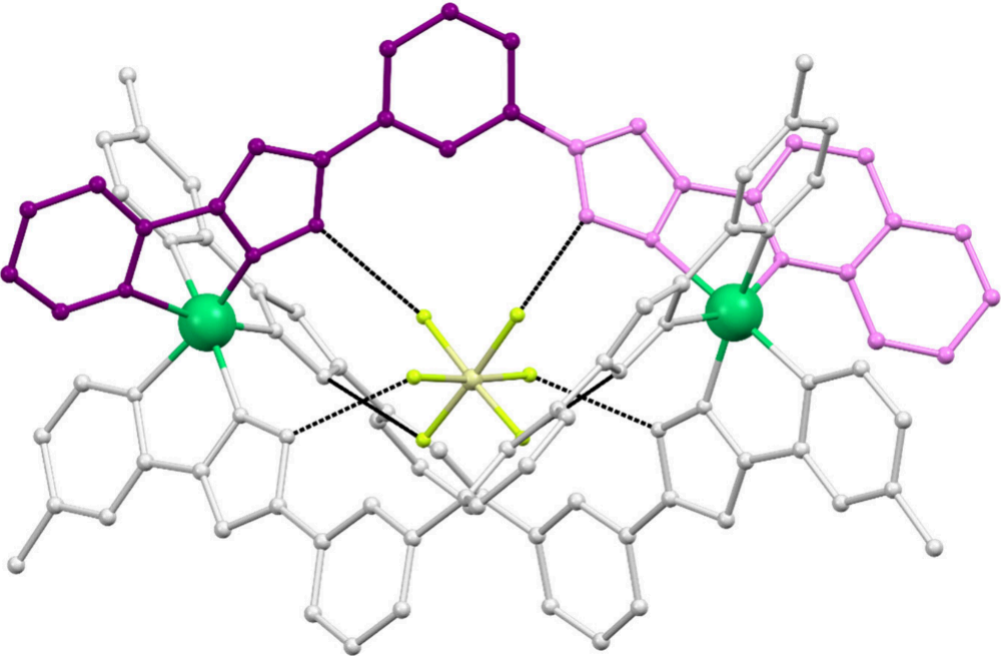
Structure of the cation (SiF_6_@[Ni_2_(L6)_2_L2])^2+^. Ligands L6 are gray whereas L2 is part dark and part light violet, emphasizing its asymmetry, which translates into the chemical (and magnetic) nonequivalence of both Ni^2+^ ions.

## 5 Control of Helicate vs Dimer Formation by Supramolecular Design

The formation of (X@[Fe_2_(L1)_3_])^3+^ helicates (X^–^ = Cl^–^, Br^–^) is in competition with the dimerization of mononuclear [Fe­(L1)_3_]^2+^ complexes yielding ([Fe­(L1)_3_]­X­[Fe­(L1)_3_])^3+^ (X^–^ = Cl^–^, Br^–^, I^–^; Figure 7), which also involves the trapping of a halide guest.^104^ In this other assembly, the ligands coordinate the metals only through one end, leaving the other end pendant. The dimer of monomers is also helical and more versatile than the dinuclear helicate, since it also incorporates I^–^, which does not fit into the latter. It is stabilized by 15 intermolecular π···π interactions, six [N–H···X^–^] hydrogen bridges and six [N–H···N] bonds (Figure 7). As the helicates do, this family of dimers exhibits SCO in the solid state, at temperatures modulated by the guest (*T*_SCO_(Cl) > *T*_SCO_(Br) > *T*_SCO_(I)).^104^

**Figure 7:**
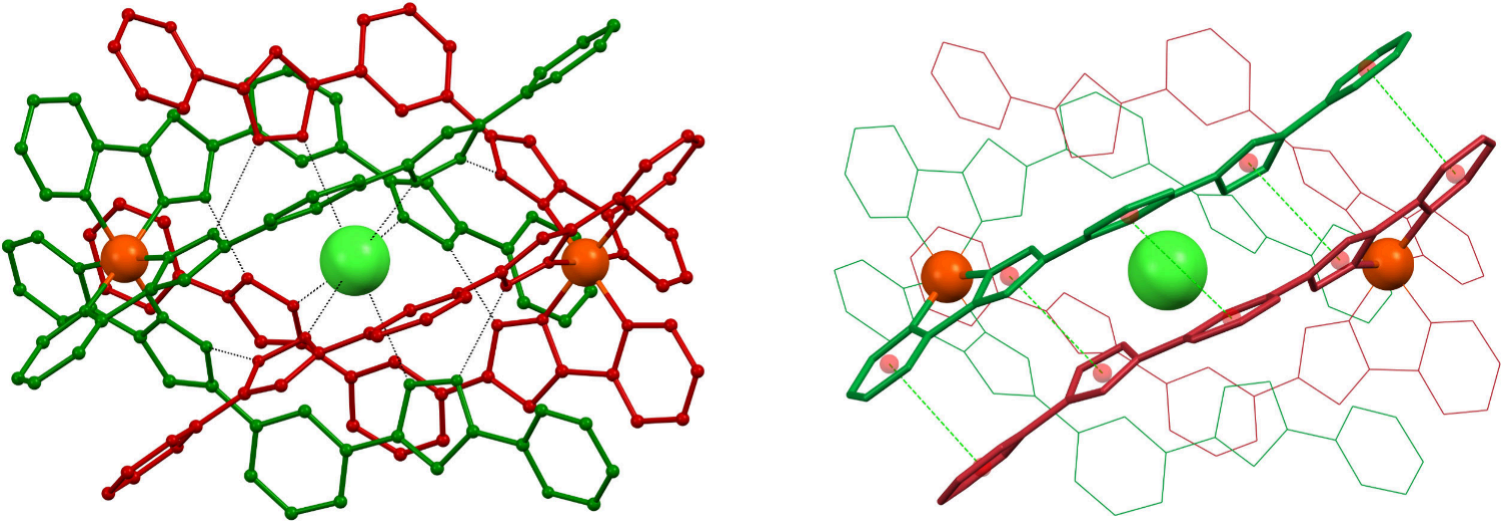
(Left) Representation of the dimer ([Fe­(L1)_3_]­X­[Fe­(L1)_3_])^3+^ (X^–^ = Cl^–^, Br^–^, I^–^). The ligands of each monomer are red and green, respectively. Fe^2+^ are orange balls, and the guest, X^–^, is green. The six hydrogen [N–H···X^–^] bonds fixing X^–^ are black dashed lines as well as the six [N–H···N] interactions. (right) The dimer emphasizing the five π···π interactions within each ligand pair (of a total of three), represented as green dashed lines linking the centroids (small red balls) of the rings involved.

^1^H NMR^89,104^ demonstrates that both types of assembly exhibit kinetic stability in solution, yet trace levels of the reciprocal species remain discernible in their respective spectra (Figure 3 for the case of the helicate). The isolation of one or the other is achieved by adjusting the reaction conditions (including the stoichiometry).^89,104^ Ligand design allows the exclusive formation of the dimer by maximizing the intermolecular interactions stabilizing it, and the tuning the magnetic properties of the assembly. Thus, L3 and L4 (Figure 2), comprising two more phenyl rings than L1, unequivocally lead to the assemblies ([M­(L)_3_]­X­[M­(L)_3_])^3+^ (L = L3, L4; M = Fe^2+^, Ni^2+^; X^–^ = Cl^–^, Br^–^, I^–^; Figure 8),^105^ which display six additional π···π interactions than the L1 counterparts. In the case of Fe^2+^, whether the ligand is quinolyl (L3) or isoquinolyl (L4) determines the magnetic state of the metal centers. Thus, the ([Fe­(L3)_3_]­X­[Fe­(L3)_3_])^3+^ assemblies are HS (for X^–^ = Cl^–^, Br^–^ and I^–^) at all temperatures whereas the L4 derivatives are LS, presumably because of the opposite steric effects caused by the position of the fused benzene ring.^106,107^ Paramagnetic ^1^H NMR spectroscopy confirms that these assemblies persists in solution and also reveals their encapsulation selectivity. The [Fe­(L3)_3_]^2+^ monomers prefer trapping Br^–^, whereas Cl^–^ is released from ([Fe­(L3)_3_]­Cl­[Fe­(L3)_3_])^3+^ as soon as it enters the solution, producing the empty dimer. Encapsulation of I^–^ is of intermediate strength; the data indicate an equilibrium between ([Fe­(L3)_3_]­I­[Fe­(L3)_3_])^3+^ and the empty dimer ([Fe­(L3)_3_]···[Fe­(L3)_3_])^4+^ together with free I^–^.^105^ The asymmetric ligand L2 (with only one extra aromatic ring compared to L1; Figure 2) also yields exclusively the dimers ([M­(L2)_3_]­X­[M­(L2)_3_])^3+^ (M = Fe^2+^, Ni^2+^; X^–^ = Cl^–^, Br^–^, I^–^; Figure 8), coordinating the metals via its pyridine end. Expectedly, the Fe^2+^ systems exhibit similar SCO in the solid state as the L1 dimers. Ligand L7 (Figure 2), with a terpyridine-like spacer in between the chelating pockets, forms with Fe^2+^ a related interdigitated dimer of [Fe­(L7)_3_]^2+^ monomers, featuring 21 π···π interactions (Figure 8) and six [N–H···N] bonds. A larger spacer allows the trapping of more and larger guests (here, two ClO_4_^–^ anions and one molecule of acetone).^101^

**Figure 8:**
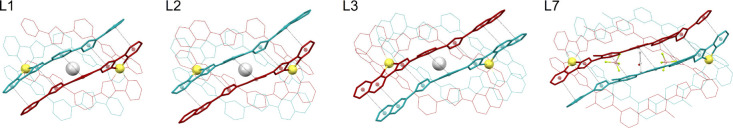
Supramolecular dimers ([M­(L)_3_]­X­[M­(L)_3_])^3+^ (L = L1, L2, L3; M = Fe^2+^, Ni^2+^; X^–^ = Cl^–^, Br^–^, I^–^) and ([Fe­(L7)_3_]­(ClO_4_)_2_(acetone)­[Fe­(L7)_3_])^2+^, for each emphasizing one of the three sets of π···π interactions essential to cement the assemblies, as dashed black lines linking the centroids of the interacting aromatic rings. In each case, the ligands from each monomer are in a different color (red or turquoise), displayed in wireframe style except for these emphasized, shown in thick stick style.

The flexibility of the ([M­(L)_3_]­(guests)­[M­(L)_3_])^*n*+^ dimers provides an entry to the encapsulation of guests with different sizes and properties establishing a versatile avenue to obtain multifunctional supramolecular assemblies.

## 6 Encapsulation of Coordination Complexes: Host-Guest Magnetic Multifunction

The biphenyl spacer separating the chelating pockets in L5 and L6 (Figure 2) generates a cavity within their corresponding [M_2_L_3_]^4+^ helicates that is suitable for the encapsulation of coordination complexes of the type [M′(ox)_3_]^3–^ (M′ = trivalent metal cation; ox = dianion oxalate; Table 1). The fit is enabled by the adequate volume, a symmetry matching that of the guest (*D*_*3*_) and the existence of six N–H hydrogen bond donor groups suitably oriented to establish six [N–H···O] interactions with the oxygen atoms of the M′–O bonds. This gives access to a family of supramolecular assemblies of the type ([M′(ox)_3_]@[M_2_L_3_])^+^ (M′ = Fe^3+^, Cr^3+^, Al^3+^; M = Fe^2+^, Ni^2+^, Co^2+^, Cu^2+^, Zn^2+^; L = L5, L6; Figure 9)^1,4,108^ with potential supramolecular multifunctionality. For example, the host of the compounds [M′(ox)_3_]@[Fe_2_(L5)_3_]­BF_4_ (M′ = Fe^3+^, Cr^3+^), with a paramagnetic guest (*S* = 5/2 for Fe and 3/2 for Cr), exhibits SCO of the Fe^2+^ ions (Table 2), opening a possibility for the mutual interaction of the magnetic properties, activated by external stimuli.^1,108^ For both compounds, the low temperature (approximately <150 K) paramagnetic response is due exclusively to the guest, since both Fe^2+^ centers of the host are diamagnetic in this regime (LS, *S* = 0). However, they are brought to a metastable HS state at low temperature when irradiated with green light, a phenomenon called light-induced excited spin state trapping (LIESST).^109^ This can be exploited to trigger magnetic synergies at low temperature with very precise time resolution, for example, to manipulate the spin dynamics of a central guest by lights via the generation of HS metastable states on the host metals.

**Figure 9:**
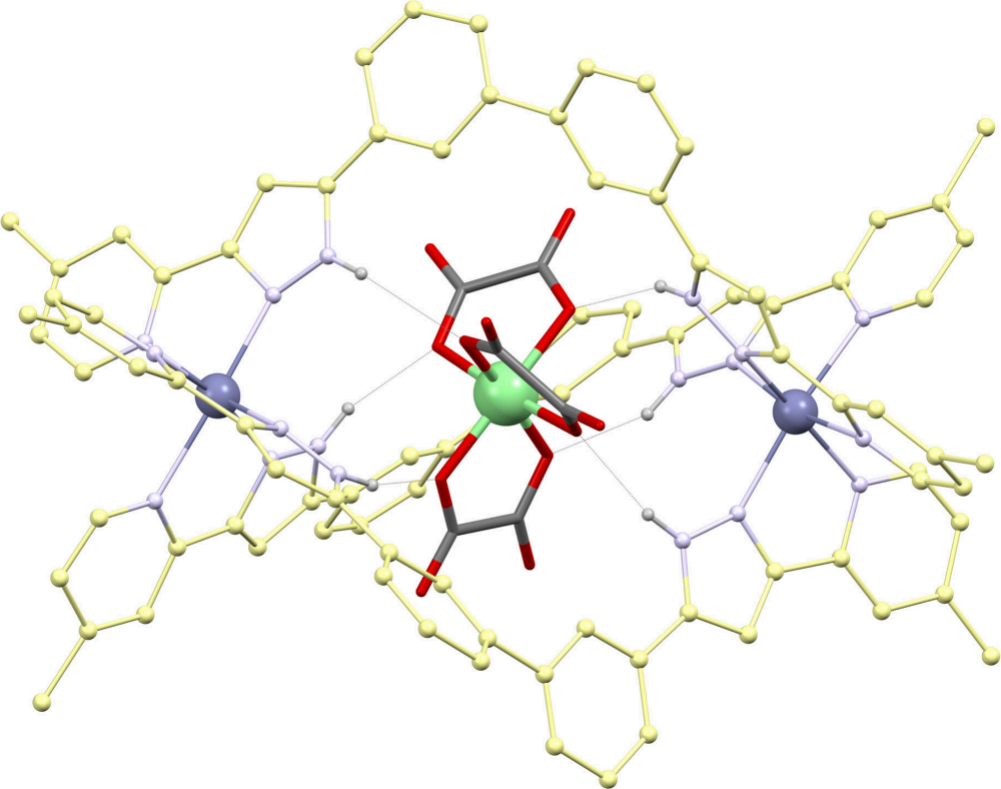
Supramolecular cation ([M′(ox)_3_]@[M_2_(L6)_3_])^+^ (M′ = Fe^3+^, Cr^3+^, Al^3+^; M = Fe^2+^, Ni^2+^, Co^2+^, Cu^2+^, Zn^2+^). Colors: C atoms from L6 are yellow and these from ox^2–^, gray; O is red, N is purple, M is blue, M′ is green, and H is light gray. Only H atoms on N–H groups are shown, with the corresponding [N–H···O] interactions emphasized with black dashed lines.

Another example of synergy is the drastic effect upon encapsulation observed on the relaxation of the magnetization of the Cr^3+^ ion. This metal ion had never been observed to exhibit SIM behavior.^110^ It was observed for the first time when the magnetic susceptibility of the compound [Cr­(ox)_3_]@[Fe_2_(L5)_3_]­BF_4_ was measured under an oscillating magnetic field.^1^ This behavior is induced when an external magnetic field, *B*, is applied. The optimal performance occurs at *ca. B* = 5000 G, yielding a relaxation time τ ≈ 0.04 ms at 1.8 K. The phenomenon was corroborated with the analogous compound [Cr­(ox)_3_]@[Zn_2_(L6)_3_]­Cl,^4^ which exhibits even longer relaxation times (up to 10 ms for *B* = 2000 G; Figure 10).

**Figure 10:**
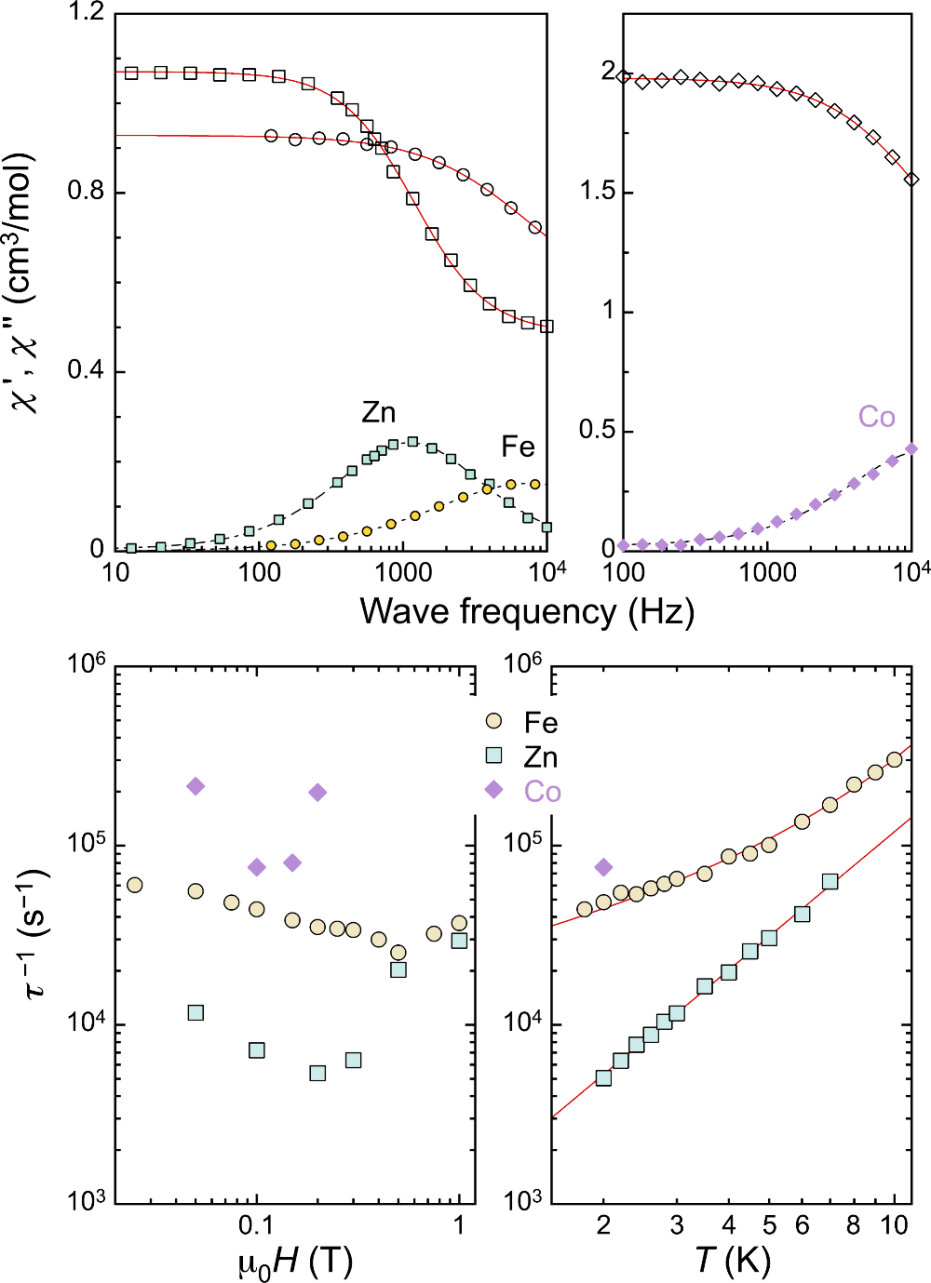
(top) Frequency dependence of the real (χ′) and imaginary (χ″) components of the ac susceptibility of [Cr­(ox)_3_]@[Fe_2_(L5)_3_]­BF_4_ (circles, *T* = 1.8 K) and [Cr­(ox)_3_]@[M_2_(L6)_3_]Cl (*T* = 2 K; M = Zn^2+^, squares; Co^2+^, rombs) under a 0.1 T dc field. Lines are fits to the generalized Debye model. (bottom) Field (left) and temperature (right) dependencies of τ^–1^ for the same three compounds, respectively, at 2 K (M^2+^ = Zn^2+^, Co^2+^) or 1.8 K (M^2+^ = Fe^2+^) and under a 0.1 T dc field (M^2+^ = Co^2+^, Fe^2+^) or 0.2 T (M^2+^ = Zn^2+^). The red lines are fits to the expressions τ^–1^ = *aT* + *bT* ^*n*^ + *c* and τ^–1^ = *bT* ^*n*^, respectively, for M^2+^ = Fe^2+^ (*a* = 1.68 × 10^4^ s^–1^, *b* = 109 s^–1^, *n* = 3.1, *c* = 1.0 × 10^4^ s^–1^) and M^2+^ = Zn^2+^ (*n* = 1.94).

SIM behavior of the host in this type of assembly is also accessible. This is the case for the compound [Al­(ox)_3_]@[Co_2_(L6)_3_]­Cl, which shows slow relaxation of the magnetization of the Co^2+^ ions (*S* = 3/2 with *g* anisotropy and zero field splitting, ZFS, of *D* = 54 cm^–1^ and *E* = 14 cm^–1^, as determined by EPR simulations), under an external *dc* field.^101^ The dependence of τ with *B* and *T* was compared with these of the related host–guest helical assemblies Cl@[Co_2_(L1)_3_])­Cl­(PF_6_)_2_, SiF_6_@[Co_2_(L1)­(L6)_2_]­(PF_6_)_2_ and ClO_4_@[Co_2_(L6)_3_]­(ClO_4_)_3_ (see above).^100^ The thermal dependence in all these systems seems dominated by Raman processes associated with the vibrations of the guest. The compound [Cr­(ox)_3_]@[Co_2_(L6)_3_]Cl provides a new example of host–guest synergy within this family of assemblies. Its magnetic relaxation is approximately 1 order of magnitude faster than that of [Al­(ox)_3_]@[Co_2_(L6)_3_]Cl (i.e., in the absence of a paramagnetic guest) and is even more strongly accelerated relative to [Cr­(ox)_3_]@[Zn_2_(L6)_3_]­Cl, in which only Cr^3+^ is magnetic. This acceleration is most likely due to the influence of the mutual host–guest magnetic interaction, which despite being almost imperceptible, has a dramatic effect on the dynamic magnetic properties.^101^

Recently, it was demonstrated that the cavity inside the [M_2_L_3_]^4+^ helicates (L = L5 and L6) can fit tris-chelate complexes of extended 1,2-diketonates such as in [M′(ani)_3_]^3–^ (M′ = trivalent metal cation; ani = anilate). Thus, the assembly [Fe­(ani)_3_]@[Fe_2_(L5)_3_]­(BF_4_) was reported,^112^ opening a new avenue for the incorporation of a wide range of functionalities. As for the related helicates, the chirality of the coordination geometry at the three metal centers is transferred to the triple-stranded helix. In this compound, the same chirality is maintained across the entire single crystal while crystals of both enantiomeric forms cocrystallize from the reaction system. In this supramolecular assembly, the guest breaks the symmetry of the host by establishing π···π interactions between its three anilate rings and three phenylene groups near one end of the host, giving rise to two magnetically inequivalent Fe^2+^ centers. Consequently, they display independent SCO behavior, which can be tracked crystallographically (Table 2). Guest-induced symmetry breaking in multifunctional helicates can thus provide a strategy to engineer site-selective magnetic responses, with implications for molecular spin control and quantum information science.

## 7 Supramolecular Enhancement of Spin Qubit Quantum Coherence

One particularly relevant functional property that can be envisioned for guests within supramolecular assemblies is their ability to function as spin-based quantum bits (qubits) for quantum technologies.^113^ In this context, it is noteworthy that encapsulation of spin qubits is emerging as a strategy for optimizing their spin dynamics and for protecting them against decoherence.^114^ The free complex [Cr­(ox)_3_]^3–^ is reported to coherently encode qubits within its *S* = 3/2 multiplet when studied in frozen solution.^115^ Remarkably, upon encapsulation within [M_2_L_3_]^4+^ helicates (L = L5 or L6), it exhibits a slower spin–lattice relaxation time (τ) as observed for the assemblies ([Cr­(ox)_3_]@[Fe_2_(L5)_3_])^+^ and ([Cr­(ox)_3_]@[Zn_2_(L6)_3_])^+^ (see above). Importantly, the structural integrity of host–guest complexes is retained upon dissolution, as confirmed by MS. This stability enables investigation of the spin dynamics of ([Cr­(ox)_3_]@[Zn_2_(L6)_3_])^+^ in solution by pulsed EPR, which shows that encapsulation reduces the quantum coherence time to approximately one-quarter of that of the free, dissolved [Cr­(ox)_3_]^3–^ qubit. By contrast, and consistent with the enhanced spin–lattice relaxation, encapsulation proves highly beneficial for quantum coherence in the solid state.^4^ This effect can be directly probed by diluting ([Cr­(ox)_3_]@[Zn_2_(L6)_3_])^+^ within an isostructural crystalline lattice made of the diamagnetic analogue ([Al­(ox)_3_]@[Zn_2_(L6)_3_])^+^_,_ thereby suppressing deleterious electron dipole–dipole interactions associated with magnetically concentrated samples. This allows the recording of echo-detected field-sweep (EDFS) pulsed EPR spectra (Figure 11). The phase memory time (*T*_*m*_) of the protected qubit measured at selected magnetic-field positions of the echo-detected EPR spectrum, reveals strong field dependence. Considering the simulation of the spectra, it is observed that the highest coherence corresponds to the intra-Kramers transition within the *m*_S_ = ± 1/2 doublet. The shorter coherence times observed at inter-Kramers transitions are consistent with enhanced spectral diffusion arising from phonon-driven modulation of the ZFS, indicative of vibronic decoherence mechanisms. The comparison of the solid-state quantum coherence with that of the free qubit was carried out on the molecular alloys ([Al_1–*x*_Cr_*x*_(ox)_3_]@[Zn_2_(L6)_3_]Cl with *x* = 0.09 and 0.03. The calculated values of *T*_*m*_ at various temperatures and magnetic fields compare favorably with these of K_3_[Al­(ox)_3_] lattices doped with [Cr­(ox)_3_]^3–^, even when the unprotected qubit is more diluted (≈ 1%). The analysis of the quantum coherence following Carr–Purcell–Meiboom–Gill (CPMG) sequences unveil a much narrower range of *T*_*m*_ values, also pointing at the interaction with phonons as the main source of decoherence. This hypothesis suggests that decoupling the qubit from lattice phonons following its encapsulation is the likely explanation of the improved solid-state dynamic properties. The ability to optimize the properties of qubits exploiting supramolecular recognition tools opens a promising pathway to advance the molecular approach as a competitive platform for the realization of qubits and qugates for quantum technologies.

**Figure 11:**
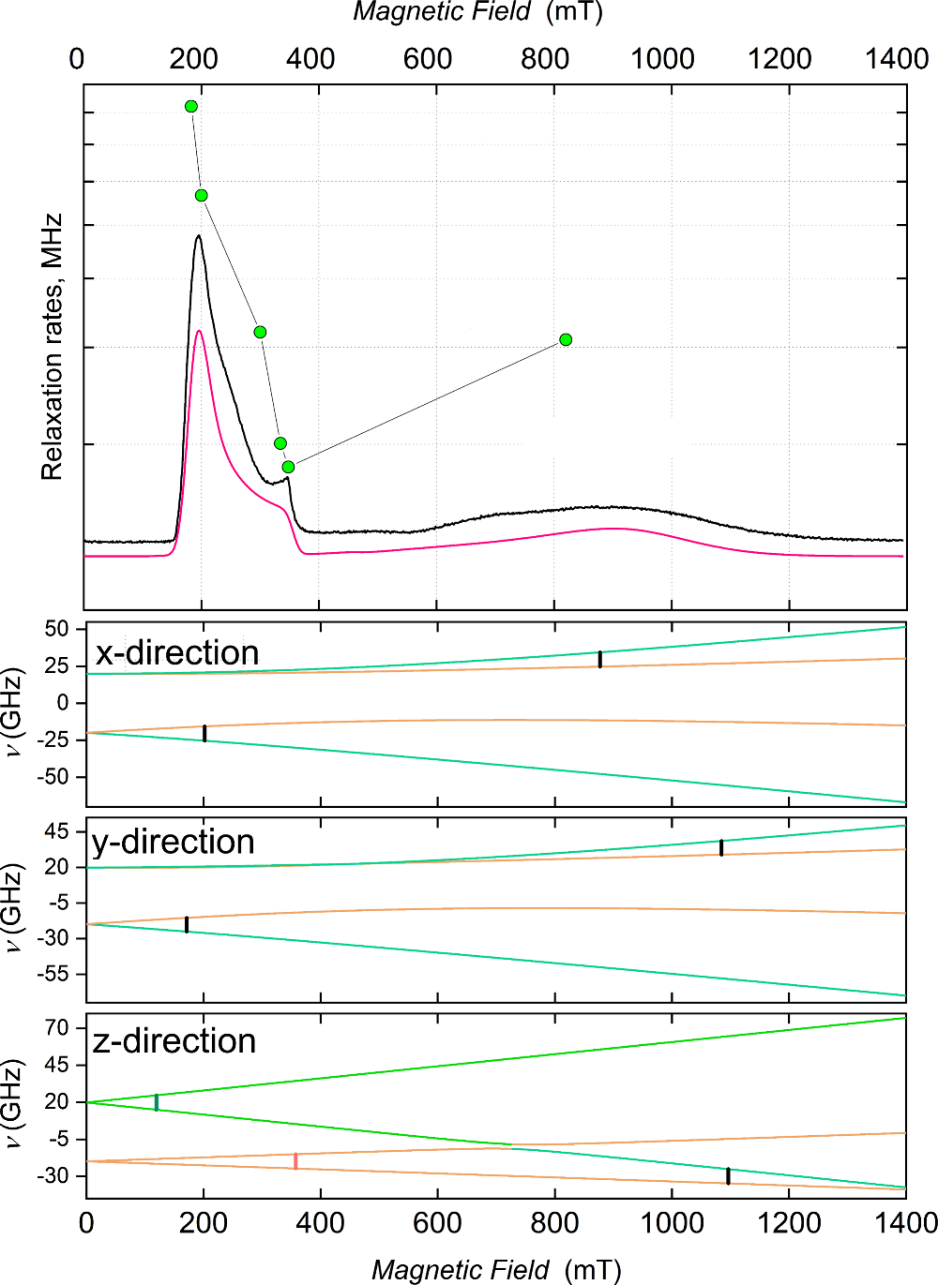
(top) X-band experimental (black) and simulated (red) EDFS pulsed EPR spectra of solid [Al_0.97_Cr_0.03_(ox)_3_]@[Zn_2_(L6)_3_]Cl at 10 K and relaxation rate values at selected magnetic fields. (bottom) Simulated energy level diagrams for the three magnetic field directions. Green and orange colors correspond to predominant *m*_S_ = ±3/2 and *m*_S_ = ±1/2 contributions, respectively. The intra-Kramers transition within the *m*_S_ = ±1/2 states is in orange; inter-Kramers transitions are in black, and the forbidden transition within *m*_S_ = ±3/2 states is in green.

## 8 Conclusions

This account surveys the ability of a class of ditopic ligands—comprising two pyrazolylpyridine or pyrazolylquinoline chelates linked by a spacer—to form triple-stranded metallohelicates capable of encapsulating anionic guests. Subtle variations in the ligands open access to a broad range of host–guest combinations. On the one hand, the host frameworks display diverse functional properties, arising from the ligand strands, the coordinated metal ions, or, occasionally, from their synergistic interplay. On the other hand, the encapsulated guests introduce a complementary spectrum of functionalities.

The examples discussed herein illustrate striking and often unprecedented synergies that emerge from the convergence of these properties within host–guest assemblies. As a result, this versatile supramolecular platform occupies a relevant position at the interface of fields relying on anion recognition, single-molecule magnetism, molecular spin-switching, and electronic spin quantum coherence. While the systems described represent a small subset of what is possible, they highlight the potential of these metallohelicates as modular and tunable architectures for future functional supramolecular systems.
